# Analysis of global research output on diabetes depression and suicide

**DOI:** 10.1186/s12991-018-0214-2

**Published:** 2018-10-23

**Authors:** Waleed M. Sweileh

**Affiliations:** Department of Physiology and Pharmacology/Toxicology, College of Medicine and Health Sciences, Nablus, Palestine

**Keywords:** Diabetes mellitus, Depression, Suicide, Research output, Bibliometric analysis

## Abstract

**Background:**

Diabetic patients, during the course of the disease, are most likely to experience depressive symptoms that might ultimately lead to suicidal ideation or suicide. The size of literature in diabetes depression/suicide is a good indicator of national and international efforts to address psychological co-morbidities associated with diabetes mellitus (DM). Therefore, the objective of this study was to give a comprehensive analysis, both quantitative and qualitative, of scientific literature in diabetes depression/suicide.

**Methods:**

SciVerse Scopus was used to retrieve relevant literature up to 2016.

**Results:**

In total, 1664 journal documents were retrieved with an average of 26.9 citations per article and an *h*-index of 98. Publications started in 1949 but showed a steep and noticeable increase after 2001. Retrieved articles were published in 641 different journals with *Diabetes Care* journal being the top productive one with a total of 130 (7.8%) articles. Researchers from 83 different countries participated in retrieved publications. Researchers from the United States of America participated in publishing 685 articles. There was a strong and positive correlation between research output and Gross Domestic Product (*r* = 0.083; *p* < 0.001) but not with prevalence or mortality caused by DM. Researchers from 4870 different institutions/organizations participated in publishing retrieved articles. Publications from the *University of Washington, Seattle, USA* had the highest *h*-index (38), while “*VA medical centers*” had the highest number of publications (75; 4.5%). In total, 5715 authors appeared in retrieved articles giving an average of 3.4 authors per article. Top cited articles focused on prevalence, impact of depression on glycemic control, and potential risk of diabetic complications. The total number of publications in depression/suicide in diabetic patients was lesser than that in cardiac (1938) or in cancer (1828) patients. However, publications in diabetes depression/suicide exceeded those in cardiac and cancer in the last 2 years of the study period.

**Conclusion:**

The current study showed a noticeable growth of publications indicative of the importance of this topic. Research focusing on the psychiatric component of diabetes mellitus needs to be strengthened and encouraged. At the practical level, screening for depression/suicide among patients attending primary healthcare clinics is needed to optimize health and quality of life of diabetic patients.

**Electronic supplementary material:**

The online version of this article (10.1186/s12991-018-0214-2) contains supplementary material, which is available to authorized users.

## Background

Diabetes mellitus (DM) is a chronic metabolic disease that requires careful changes in life style that can be demanding and difficult to implement by some diabetic patients [1]. According to World Health Organization’s (WHO) recent report, the number of people diagnosed with diabetes mellitus (DM) has risen from 108 million in 1980 to 422 million in 2014 [2]. In 2012, an estimated 1.5 million deaths were directly caused by diabetes and another 2.2 million deaths were attributable to high blood glucose [2]. Diabetes mellitus is considered as a national and global health burden. It is estimated that at least 10% of healthcare expenditures in many countries is invested in preventing and combating DM complications [3]. Diabetes mellitus is not only a health and economic burden but also a social and psychological challenge that could ultimately lead to chronic depression.

Depression is a common mental disorder and according to the WHO, more than 300 million people worldwide had depression. Depression is a serious illness and if not properly addressed, it can affect the normal function of affected people and might sometimes lead to suicide [4]. Globally, there is an increased trend of suicide [5]. According to the WHO, close to 800,000 deaths occur annually due to suicide and the majority (78%) of these cases occur in low- and middle-income countries (LMIC) [6]. Psychiatric disorders are known to impair the control of chronic diseases such as DM and behavioral interventions in such conditions might have more pronounced effects than medications [7].

Diabetic patients, and during the course of the disease, are most likely to experience depressive symptoms that might ultimately lead to suicidal ideation or suicide. Published studies showed that individuals with diabetes have an increased incidence of major depression when compared to the general population [8–10]. The high prevalence of depression among diabetic patients had led to the term “diapression” [11]. Vascular changes due to DM could be the biological basis for the development of depression among diabetic patients [12]. The relationship between diabetes and depression could be bidirectional with one disease leading to the increased risk of having the other disease [13]. Regardless of the directionality of the disease, the presence of depression in diabetes mellitus could worsen self-care, poor medication adherence, increased healthcare cost, poor glycemic control, potential risk of diabetic micro- and macro-vascular complications, and poor QOL [14–16]. The presence of depression in diabetic patients could lead to suicide ideation and suicide attempts. Studies have shown that diabetic patients, particularly type 1 DM, have higher risk of suicide ideation and suicidal attempts than non-diabetic patients [17, 18].

In light of increasing incidence of diabetes mellitus and in light of geographical and social differences in healthcare services and health literacy in diabetes, the need to assess the growth of research on diabetes depression/suicide becomes very important. Quantitative and qualitative analysis of publications in a particular area is usually called bibliometrics or scientometrics in which statistical methods are applied on a set of retrieved publications [19]. Bibliometric analysis is a growing field of information science which had been applied to various scientific disciplines [20–24]. Bibliometric analysis is a key element in establishing baseline data for future comparison in any scientific subject. Bibliometric analysis in diabetes depression/suicide could help to establish strategies for improving the volume and quality of research in this field and the results could help to identify research gaps that future studies could focus on. The size of literature and research productivity in diabetes depression/suicide are good indicators of national and international efforts to decrease the health and economic burden of DM and the national and international efforts to address psychological co-morbidities associated with diabetes that could affect the QOL and glycemic control in diabetic patients.

To date, no studies have been published to summarize global research efforts, research trends, and geographical distribution of research output in diabetes depression/suicide, despite that several bibliometric analyses in diabetes research activity had been published [21, 25–28]. Therefore, the objective of this study was to give a comprehensive analysis, both quantitative and qualitative, of scientific literature in diabetes depression/suicide.

## Methods

### Bibliographic database

For the purpose of this study, only peer-reviewed articles published in scientific journals indexed in SciVerse Scopus were retrieved. Gray literature such as governmental and non-governmental reports, brochures, dissertations, theses, and newsletters were not included because some of the gray literature especially thesis and dissertation might have been published as research articles in peer-reviewed journals and therefore, they will create false-positive results due to overlap. The choice of Scopus database was based on the understanding of the author that it is larger than Web of Science and includes 100% of Medline [29]. Furthermore, Scopus has many analytic functions that facilitate bibliometric investigations of retrieved literature and therefore, it had been used in many previously published articles in the medical field.

### Research strategy and keywords

To achieve the goal of the study, a set of related keywords pertaining or indicative of diabetes along with keywords related to depression or suicide were used. Keywords used in the search strategy were obtained from available systematic reviews [30–35]. The search strategy and keywords used along with the number of documents retrieved in each step are shown in Additional file 1: Appendix S1. To avoid any misinterpretation, we excluded publications in gestational diabetes mellitus and in experimental animals. The search strategy was based on searching for specific keywords in title of articles and not in abstract or author keywords. Actually, search for keywords in abstract and/or keywords yielded too many false-positive results that could negatively affect the validity of the study.

### Validity check

The validity of research strategy was checked by manual review of top 20 cited articles to guarantee the absence of false-positive results. Furthermore, visualized author keywords were used to check for any irrelevant terms or false-positive results. For example, it was noticed that some keywords such as neuropathic pain, diabetes insipidus were present in retrieved articles and therefore, such keywords were excluded. Finally, research productivity of top active authors was retrieved manually and compared with those obtained using the current search strategy. The correlation between the manually obtained results and those obtained by search strategy was high with an interclass correlation of 96.8% indicative of high validity of the results and very low percentage of false negative.

### Bibliometric indicators

Retrieved documents were refined, analyzed, and mapped to show research contribution and research trends. The time span of the study was set from 1997 to 2016. The Hirsch index (*h*-index) was used to assess the quality of published literature, while impact factor (IF) obtained from Journal Citation Report (2016) was used to assess the strength of publishing journals. Analysis of growth of publications with time was presented graphically using Statistical Package for Social Sciences (SPSS 21). For mapping keywords as well as international research collaboration, VOSviewer software was used [36]. In VOSviewer, the extent of collaboration is assessed by the thickness of a line connecting any two items such as countries or authors. For research productivity, the larger circle size or font size presenting a country or author, the greater the research productivity or citations of the listed author or country [36]. For geographical distribution of publications, ArcMap 10.1 software was used. Active institutions/organizations as well as most preferred journals for publishing articles in DSM were presented as top ten ones.

## Results

The total number of retrieved articles was 1664. Research articles constituted the majority (1311; 78.8%) of retrieved documents followed by review articles (160; 9.6%) (Table 1). The main language in retrieved articles was English (1497; 90.0%) followed by German (67; 4.0%) and Spanish (30; 1.8%). Retrieved documents had an *h*-index of 98 and the highest number of citations recorded was 2060 for a meta-analysis study published in 2001 about the prevalence of depression among diabetic patients [37]. Retrieved documents received a total number of citations of 44,775, an average of 26.9 citations per article. The vast majority of retrieved articles was about diabetes depression, while 46 (2.8%) articles were about diabetes suicide and suicidal ideation.

**Table 1:** Types of retrieved documents

Document type	Frequency	% (*N* = 1664)
Article	1311	78.8
Review	160	9.6
Letter	63	3.8
Note	57	3.4
Conference paper	22	1.3
Editorial	19	1.1
Short Survey	18	1.1
Article in Press	14	0.8

Of retrieved articles, 765 (46%) were in the subject area of “Medicine”, 311 (18.7%) were in the subject area of “Biochemistry, genetics, immunology, and molecular biology”, while 227 (13.6%) were in the subject area of “psychology”. Various subject areas of retrieved articles are shown in Table 2.

**Table 2:** Subject areas of retrieved documents

Document type	Frequency	% (*N* = 1664)^a^
Medicine (General and Internal)	765	46.0
Biochemistry, Genetics and immunology, Biology, and Molecular Biology	311	18.7
Psychology	227	13.6
Nursing	220	13.2
Neuroscience	80	4.8
Social Sciences and Humanities	92	5.5
Pharmacology, Toxicology and Pharmaceutics	47	2.8
Health Professions	41	2.5
Multidisciplinary and Miscellaneous	17	1.0

^a^Total percentage exceeds 100% due to overlap in certain subject areas

**Fig. 1:**
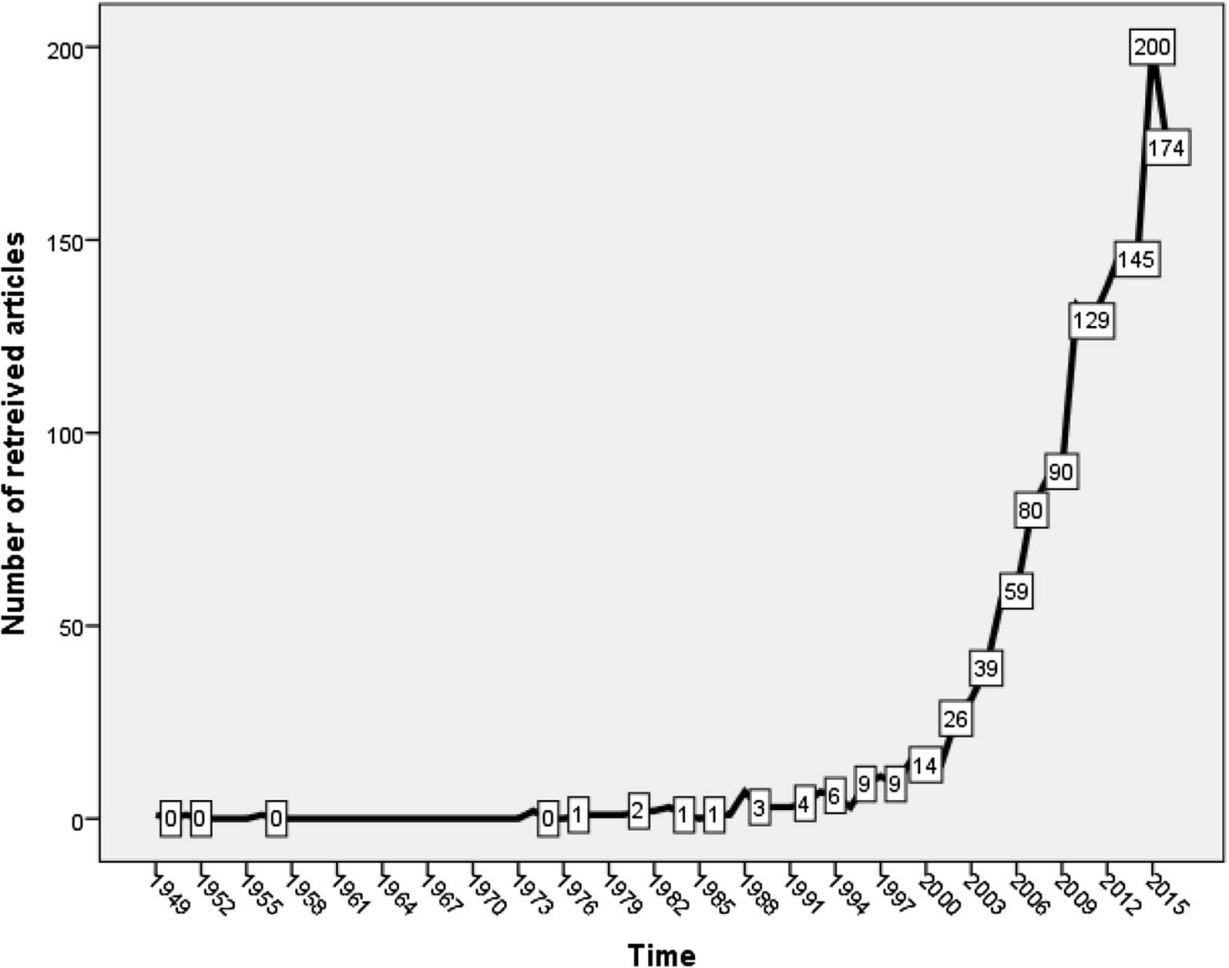
Growth of publications in diabetes depression/suicide

Growth of publications in diabetes depression/suicide started in 1949 with an article published in *The New England Journal of Medicine* about the effects of a large dose of insulin taken for suicidal attempt [38]. The number of publications in diabetes depression/suicide started in 1949, but remained very low until 2001. After 2001, the number of publications showed a steep and noticeable increase. Figure 1 shows the annual growth of publications in diabetes depression/suicide. Approximately, 92% of retrieved articles were published during the period from 2001 to 2016. The remaining 8% were published from 1949 to 2001. The highest number of publications recorded was 200 publications obtained in 2015.

**Fig. 2:**
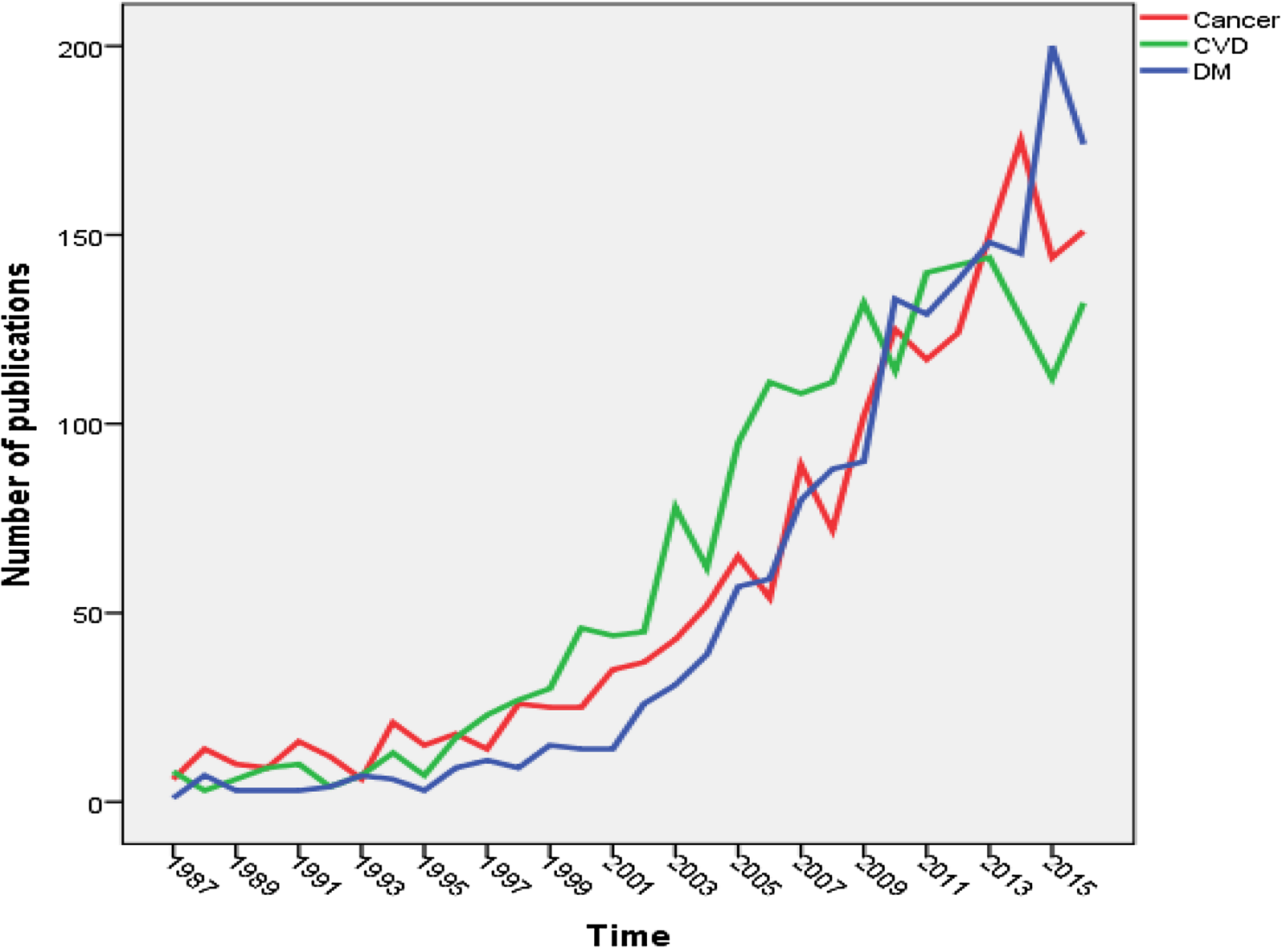
Comparison of growth of publications in diabetes depression/suicide with that in cardiac diseases and in cancer (1986–2016). DM, diabetes mellitus; CVD, cardiovascular diseases

Growth of publications was compared with that in other chronic diseases, particularly cancer and cardiac diseases. The total number of publications in diabetes depression/suicide (1664) was lesser than that in cardiac diseases depression/suicide (1938) and lesser than that in cancer depression/suicide (1828). However, the growth of publications in the three diseases showed that the ones in diabetes depression/suicide exceeded the other two in the last 2 years of the study period. Figure 2 showed the growth of publications in the three diseases with focus on the past three decades to facilitate comparison of growth of publications.

Retrieved articles were published in 641 different journals. Names of journals that published at least 10 documents are shown in Table 3 along with their most recent IF. The active list included a total of 18 journals with *Diabetes Care* being the top productive one with a total of 130 (7.8%) articles. More than half (10; 55.6%) of top active journals were in the field of diabetes and six were in the field of psychiatry/psychology (6; 33.3%), one (*Plos One*) was multidisciplinary, and one was in internal medicine. Seventeen journals in the active list had an official IF and two had an IF above five.

**Table 3:** List of journals with a minimum of 10 publications in diabetes depression/suicide

*Journal*	Frequency	% (*N* = 1664)	IF	Publisher
*Diabetes Care*	130	7.8	11.9	ADA
*Diabetic Medicine*	69	4.1	3.054	Wiley
*Diabetes Research and Clinical Practice*	39	2.3	3.693	Elsevier
*General Hospital Psychiatry*	29	1.7	2.279	Elsevier
*Journal of Affective Disorders*	28	1.7	3.432	Elsevier
*Journal of Psychosomatic Research*	26	1.6	2.809	Elsevier
*Psychosomatic Medicine*	25	1.5	3.863	LWW
*Diabetologia*	24	1.4	6.080	Springer Berlin Heidelberg
*Diabetologe*	23	1.4	0.072	Springer Medizin
*Plos One*	22	1.3	2.806	Public Library of Science
*Journal of Diabetes and Its Complications*	21	1.3	2.734	Elsevier
*Diabetes Educator*	17	1.0	1.811	Sage
*Psychosomatics*	17	1.0	1.436	Elsevier
*BMC Psychiatry*	16	1.0	2.613	BioMed Central
*Journal of General Internal Medicine*	13	0.8	3.701	Springer US
*Journal of The Japan Diabetes Society*	11	0.7	N/A	Japan Diabetes Society
*Current Diabetes Reports*	10	0.6	3.387	Current Medicine Group
*Diabetes Spectrum*	10	0.6	N/A	ADA

IF, impact factor; ADA, American Diabetes Association

**Fig. 3:**
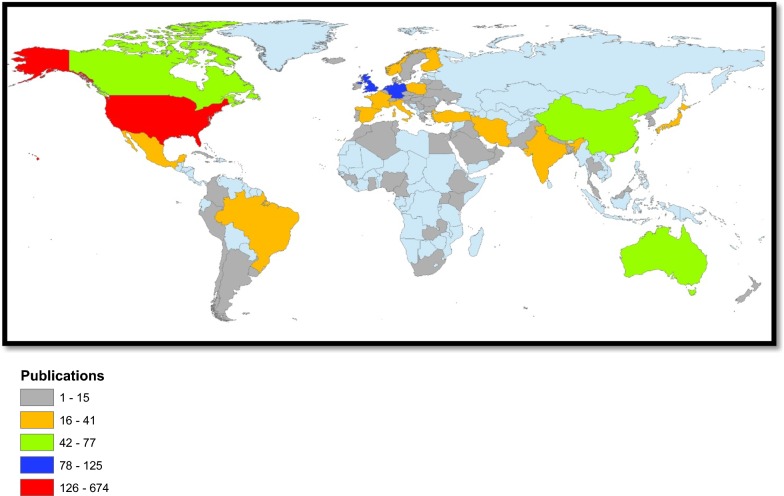
Geographical distribution of publications in diabetes depression/suicide. No data were available from regions with light blue color

Researchers from 83 different countries participated in publishing retrieved articles. Countries with a minimum productivity of 10 documents are listed in Table 4 along with the reported prevalence of DM in each country. No significant correlation existed between prevalence of DM and research productivity in diabetes depression/suicide. The most productive country was the United States of America (USA) with a total productivity of 685 documents followed by the United Kingdom (UK) with a total productivity of 125 documents. No significant correlation was found between research output and prevalence of DM or mortality caused by DM per 100,000 population. However, there was a strong and positive correlation between research output and GDP (*r* = 0.083; *p* < 0.001). Despite that there was no significant correlation between research output and mortality rate caused by DM, there was a general trend of increased mortality caused by DM with low research output. This trend was obvious in countries such as Oman, Bahrain, Jordan, Morocco, South Africa, and Mexico (data not shown). Geographical distribution of publications showed that Africa, East Europe, and South America had poor research output in the field of diabetes depression/suicide. Figure 3 is a world map for geographical distribution of publications.

**Table 4:** List of countries with minimum participation of 10 articles in diabetes depression/suicide based on country affiliation of authors

Country	Frequency	% (*N* = 1664)	Prevalence of DM [93]	Mortality per 100,000 population caused by DM [94]	GDP (trillions)
United States	674	40.5	9.1%	14.78	18.04
United Kingdom	125	7.5	7.7%	4.91	2.86
Germany	121	7.3	7.4%	11.43	3.36
Netherlands	100	6.0	6.1%	8.99	0.75
Australia	77	4.6	7.3%	11.05	1.2
Canada	71	4.3	7.2%	11.01	1.55
China	57	3.4	9.4%	14.8	11.06
India	41	2.5	7.8%	25.4	2.1
Japan	37	2.2	10.1%	4.37	4.38
Brazil	35	2.1	8.1%	39.74	1.8
Italy	31	1.9	8.5%	13.13	1.82
Poland	31	1.9	9.5%	10.07	0.477
Iran	25	1.5	10.3%	16.34	0.425
Mexico	23	1.4	10.4%	89.56	1.14
Turkey	23	1.4	13.2%	12.61	0.717
France	21	1.3	8.0%	8.78	2.42
Taiwan	21	1.3	–	–	1.177
Norway	19	1.1	6.6%	8.64	0.387
Spain	19	1.1	9.4%	9.86	1.19
Finland	17	1.0	7.7%	4.66	0.232
South Korea	15	0.9	9.5%	16.01	1.378
Sweden	14	0.8	6.9%	9.68	0.496
Austria	13	0.8	6.0%	15.84	0.377
Switzerland	13	0.8	5.6%	7.67	0.671
Saudi Arabia	12	0.7	14.4%	35.61	0.646
Croatia	11	0.7	9.9%	14.04	0.049
Hong Kong	11	0.7	–	–	0.309
Nigeria	11	0.7	4.3%	42.95	0.877
Belgium	10	0.6	6.4%	8.03	0.455
Pakistan	10	0.6	9.8%	39.65	0.271

Researchers from 4870 different institutions/organizations participated in publishing retrieved articles. Institutions/organizations with a minimum productivity of 10 documents were shown in Table 5. The active top 10 list included seven institutions in the USA, two in Netherlands and one in Canada. Publications from the *University of Washington, Seattle, USA* had the highest *h*-index (38) while “VA medical centers” had the highest number of publications (75; 4.5%).

**Table 5:** List of top active institutions/organizations

Institution/Organization	Frequency	% (*N* = 1664)	*h*-index of the publications	Country
VA Medical Center	75	4.5	31	USA
University of Washington, Seattle	73	4.4	38	USA
Tilburg University	51	3.1	22	Netherlands
VU University Medical Center	43	2.6	22	Netherlands
Johns Hopkins University	39	2.3	23	USA
Washington University in St. Louis, School of Medicine	37	2.2	26	USA
University Michigan Ann Arbor	34	2.0	15	USA
McGill University	31	1.9	8	Canada
Harvard Medical School	31	1.9	16	USA
University of California, Los Angeles	30	1.8	14	USA

**Fig. 4:**
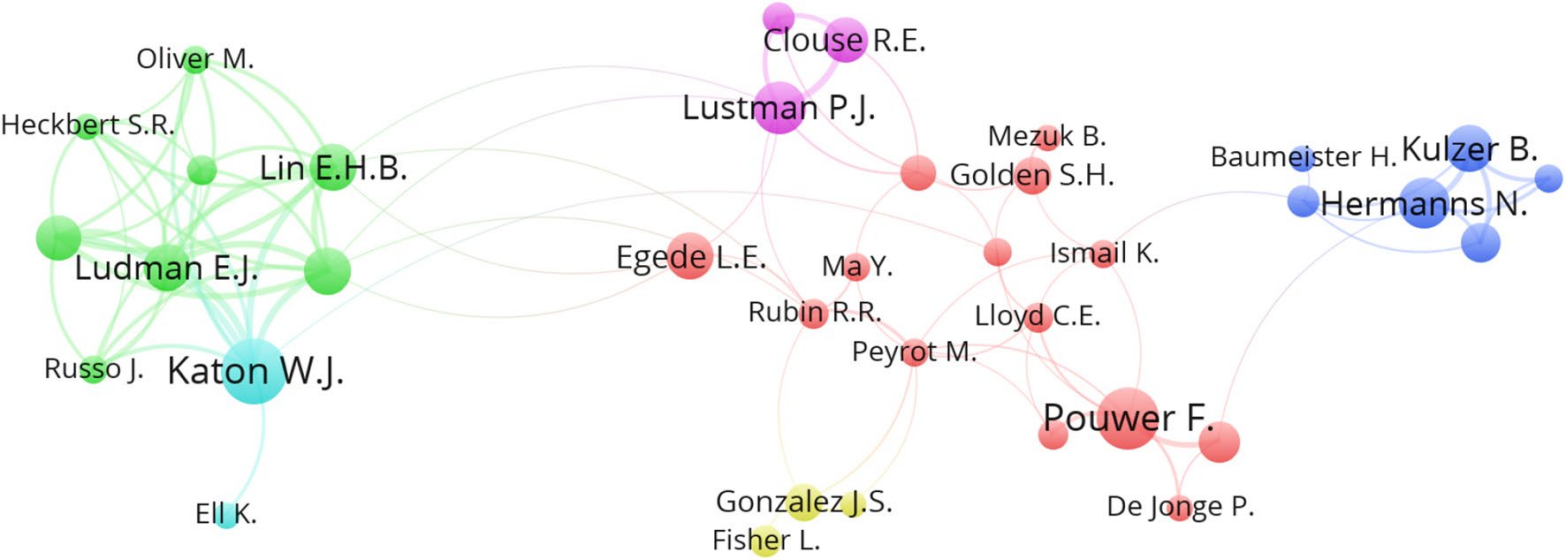
Network visualization map of authors

In total, 5715 authors participated in publishing the retrieved documents giving an average of 3.4 authors per article. Authors with a minimum productivity of 10 documents and belong to a network of authors are visualized in Fig. 4. Visualization map of active authors included 36 authors grouped into six clusters. The largest cluster (red cluster) included 14 authors forming a network of collaboration. The size of the circle in the map reflects the size of the productivity, while the lines connecting the circles reflect the extent of author collaboration. Authors with the highest productivity include Katon W.J., Pouwer F., and Lustman P.J.

**Fig. 5:**
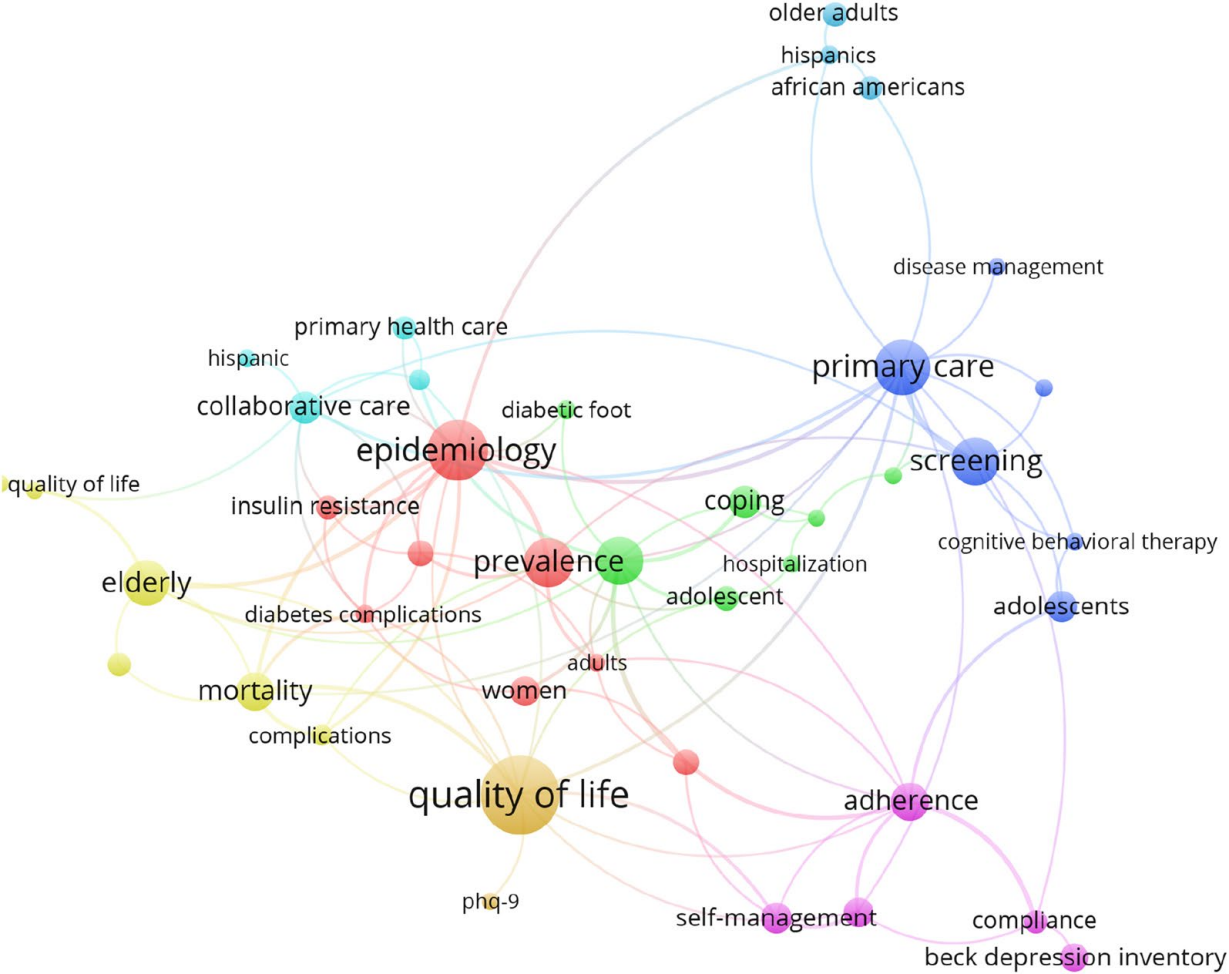
Network visualization map of relevant author keywords

Top 20 cited articles are listed in Table 6. Top cited articles discussed issues related to epidemiology, impact of depression on glycemic control, diabetic complications/mortality, medication adherence, and quality of life. Four articles in top 20 cited articles discussed depression as a risk factor for diabetes and the bidirectional relationship between diabetes and depression. Analysis of author keywords using VOSviewer mapping showed that the following author keywords were most frequently encountered: “quality of life”, epidemiology, prevalence, complications, screening, “primary care”, adherence, self-management, mortality, women, “African–Americans”, Hispanics, and other related terms (Fig. 5).

**Table 6:** Top 20 cited articles in diabetes depression/suicide

Title	References	Year	Source title	Cited by
The prevalence of comorbid depression in adults with diabetes: a meta-analysis	[37]	2001	Diabetes Care	2056
Depression and poor glycemic control: a meta-analytic review of the literature	[95]	2000	Diabetes Care	1047
Association of depression and diabetes complications: a meta-analysis	[96]	2001	Psychosomatic Medicine	987
Depression and diabetes: impact of depressive symptoms on adherence, function, and costs	[97]	2000	Archives of Internal Medicine	935
Depression and type 2 diabetes over the lifespan: a meta-analysis	[40]	2008	Diabetes Care	572
The prevalence of co-morbid depression in adults with Type 2 diabetes: a systematic review and meta-analysis	[39]	2006	Diabetic Medicine	537
Relationship of depression and diabetes self-care, medication adherence, and preventive care	[98]	2004	Diabetes Care	528
Prevalence of depression in adults with diabetes: an epidemiological evaluation	[99]	1993	Diabetes Care	480
The pathways study: a randomized trial of collaborative care in patients with diabetes and depression	[100]	2004	Archives of General Psychiatry	475
Depression as a risk factor for the onset of type 2 diabetes mellitus. a meta-analysis	[101]	2006	Diabetologia	441
Examining a bidirectional association between depressive symptoms and diabetes	[102]	2008	JAMA—Journal of the American Medical Association	432
Comorbid depression is associated with increased health care use and expenditures in individuals with diabetes	[103]	2002	Diabetes Care	426
Depression and risk for onset of type ii diabetes: a prospective population-based study	[104]	1996	Diabetes Care	417
Relationship of depression to diabetes types 1 and 2: epidemiology, biology, and treatment	[105]	2003	Biological Psychiatry	412
Cognitive behavior therapy for depression in type 2 diabetes mellitus. A randomized, controlled trial	[106]	1998	Annals of Internal Medicine	395
The association of comorbid depression with mortality in patients with type 2 diabetes	[107]	2005	Diabetes Care	371
Depression and diabetes treatment nonadherence: a meta-analysis	[108]	2008	Diabetes Care	347
Depression predicts increased incidence of adverse health outcomes in older Mexican Americans with type 2 diabetes	[109]	2003	Diabetes Care	338
Levels and risks of depression and anxiety symptomatology among diabetic adults	[110]	1997	Diabetes Care	321
Diabetes, depression, and quality of life: a population study	[111]	2004	Diabetes Care	314

## Discussion

The purpose of this study was to assess and analyze global research output and research trends on diabetes depression/suicide. The current study was conducted based on the assumption that diabetes mellitus is a potential risk factor for depressive symptoms which could lead to suicidal ideation and death [39–41]. The current study showed that keywords such as screening and “primary healthcare” were most commonly encountered indicating that screening for depression primary healthcare centers is strongly advocated by researchers.

### Subject areas of the retrieved literature

The current study showed that approximately 20% of publication in diabetes depression/suicide was within biochemistry/genetics/molecular biology subject area indicating that there was intensive research in the biological relationship between diabetes and depression. The hypothalamic–pituitary–adrenal (HPA) axis, cortisol, increased catecholamine, and proinflammatory cytokine levels play a key role in development of insulin resistance [12, 42, 43]. The current study also showed that approximately 14% of retrieved documents were within the subject area of psychology. This was unsurprising given that coping behaviors and social support for diabetic patients might be different among different cultures and ethnicities [44–54].

### Growth of publications

Research in diabetes depression/suicide started more than six decades ago, but the number of publications accelerated and showed a dramatic increase in the past decade. The growth of publication could be due to several factors. The overall increase in number of scholars, institutions, peer-reviewed journals, and global research output in the field of medicine positively affected the number of publications in diabetes depression/suicide. Second, certain findings regarding the scientific link between depression and poor glycemic control have triggered more research in this field. A third potential reason for the growth of publications was the debate about the bidirectional relationship between diabetes and depression and the role of depression as a potential risk factor for diabetes [13, 55–59]. The growth of publications is of great relevance to mental health practitioners and should argue in favor of routine depression screening in diabetic patients in primary healthcare facilities [60–68].

### Comparison with other diseases

The current study showed that the growth of research on diabetes depression showed a steeper increase than that on cardiac or cancer-depression research. Actually, in the last 3 years of the study period, the number of publications in diabetes depression/suicide exceeded that of cardiac or cancer publications indicating a global interest in this field. Furthermore, *h*-index of publications related to diabetes depression was very close to that of cardiac and cancer suggesting that depression in all chronic diseases is a serious problem that is of interest to scholars.

### Geographical distribution of publications

The current study showed that geographical distribution of publications was skewed toward developed countries, particularly USA, Canada, Australia, Germany, Netherlands, and UK. Other countries such as Brazil, India, and China were among top active countries. In 2000, India had the highest number of people with diabetes mellitus followed by China and the United States. It is predicted that by 2030 diabetes mellitus may afflict up to 79.4 million, 42.3 million, and 30.3 million individuals in India, China, and the USA, respectively, in 2030 [69–71]. Unfortunately, diabetes has been rising more rapidly in MLIC which necessitates more rigorous research in these countries to minimize diabetic complications and mortality [2]. Previously published studies also showed that contribution of LMIC to diabetes research did not match the health and economic burden of diabetes in these countries [21, 25, 27, 28, 72–74]. Not only diabetes research but also mental health research in LMIC was reported to be scarce [75–77]. The low research productivity from LMIC was also reflected in top 10 active institutions in diabetes depression/suicide research where no institution from LMIC showed up in the list.

### Highly cited documents

An important aspect discussed in the top cited documents was the impact of depression on medication adherence and the potential risk of poor glycemic control. Studies showed that co-morbid diabetes and depression decreased the likelihood of adherence to lifestyle changes, specifically, diet, medication adherence, and physical activity resulting in elevated HbA1c and consequently poor self-care/self-management with increased risk of retinopathy, nephropathy, cardiac dysfunction, and mortality [78–84]. It had been found that depressive disorders decrease the desire to seek treatment making depression as an initial step of poor diabetes outcomes [85].

#### Limitations

The current study, like other bibliometric studies, has limitations that are inherent to bibliometric methodology and nature the database used [86–92]. Scopus database is not 100% comprehensive of literature, and therefore, some literature was missed particularly the ones published in un-indexed journals from developing countries. Second, the potential presence of false-negative results is a possibility due to the use of title search strategy. However, the title research strategy was used to minimize the false-positive results.

## Conclusion

The current study showed an increasing interest of researchers in the psychiatric aspects of diabetes. This increasing interest is believed to promote the health of diabetic patients through initial screening of depression and through psychological and pharmacological treatment of the diseases. As a chronic disease with increasing global health burden, researchers need to get involved in all aspects that can alleviate the future complications of the disease to minimize health and economic burden of the disease. Future studies should focus on both epidemiological aspects in various cultures in developing countries and on the biological basis of depression in diabetic patients.

## Additional file

**Additional file 1: Appendix S1.** Global research output in diabetes depression and suicide.

## Abbreviation

DM: diabetes mellitus
WHO: World Health Organization
