# Supplementary material for: Analysis of global research output on diabetes depression and suicide
Source: Ann Gen Psychiatry. 2018 Oct 23;17:44. doi: 10.1186/s12991-018-0214-2 (PMC6198430; doi:10.1186/s12991-018-0214-2)
Supplement: Supplementary file 1 — Additional file 1: Appendix S1. Global research output in diabetes depression and suicide. [file 12991_2018_214_MOESM1_ESM.docx]

**Global Research Output in Diabetes Depression and Suicide**

**Appendix 1**

**Diabetes mellitus**: **TITLE** (diabetic OR diabetes OR "type 2 dm" OR "type 1 dm" OR t2dm OR t1dm OR "glycemic control" OR "glycaemic control" OR "elevated blood glucose" OR "elevated blood sugar")

**AND**

**Depression/suicide:** **TITLE** ("depressive symptom*" OR "depressive state*" OR "depressive disorder" OR suicid* OR depression OR depressed) OR ( TITLE ( "mood disorder*") AND ABS (depressed OR depression) ) AND (ALL(diabetes AND depression) OR ALL(diabetes AND suicid*)))

**AND NOT**

**TITLE** ( rabbit OR "neuropath* pain" OR "gestation*" OR pregnancy OR insipidus OR rats OR rat OR animal OR dog OR cat OR "cell*" OR signal* OR rodent* OR "new agents" OR mice OR alcohol* OR "non-diabet*" OR "manic" OR sleep* OR ST OR S-T OR cardiac OR myocardial OR coffee OR bipolar OR manic-depressive OR streptozo* OR renal OR neuropathy OR sensorium OR retinal OR cerebral OR parent* OR sympathetic* OR STZ OR mother* OR maternal OR "c-reactive" OR hearing OR "Na+/Ca2+ exchanger" OR "depressed heart" OR "depressed cardiac" OR interferon* OR metallo* OR "diabetes educators") )))

**AND**

(LIMIT-TO (SRCTYPE,"j ") ) AND ( EXCLUDE ( DOCTYPE,"er " ) ) AND ( EXCLUDE ( PUBYEAR,2017 ) )
